# Effect of portal vein embolisation on the growth rate of colorectal liver metastases

**DOI:** 10.1038/sj.bjc.6604872

**Published:** 2009-02-10

**Authors:** V Pamecha, A Levene, F Grillo, N Woodward, A Dhillon, B R Davidson

**Affiliations:** 1 Department of Hepato Pancreatico Biliary and Liver Transplant Surgery, Royal Free Hospital, University College London, London, UK; 2 Department of Pathology, Royal Free Hospital, University College London, London, UK; 3 Department of Radiology, Royal Free Hospital, University College London, London, UK

**Keywords:** portal vein embolisation, colorectal liver metastases, liver resection

## Abstract

Portal vein embolisation (PVE) is used to increase the remnant liver volume before major liver resection for colorectal metastases. The resection rate after PVE is 60–70%, mainly limited by disease progression. The effect of PVE on tumour growth rate has not been investigated. The objective of this study was to compare the growth characteristics of resected colorectal liver metastases in patients undergoing pre-operative PVE with those of matched controls who had not undergone PVE. There were 22 patients who had undergone preoperative PVE and 20 matched controls. Tumour growth rate was calculated by the change in tumour volume (CT/MRI volumetric assessment) from diagnosis to resection. Resected histological specimens were examined by two histopathologists independently for cell differentiation, percentage tumour cell necrosis and mitotic rate. Immunochemical staining with *Ki67* was carried out using the MIB-1 monoclonal antibody and quantified using a Glasgow cell-counting graticule. The groups were comparable in demographics, stage of primary disease, volume of liver metastases at presentation and chemotherapy received. The tumour growth rate calculated from imaging was more rapid in the PVE group compared with that in controls (control: 0.05±0.25 ml day^−1^, PVE: 0.36±0.68 ml day^−1^, *P*=0.06). Histology showed no difference in the degree of differentiation, extent of necrosis or apoptosis between the two groups. However, mitotic rate was higher post PVE, as was the proliferation index *Ki67* (*P*=0.04). This study has confirmed that tumour growth rate increased following PVE and that this is related to increased tumour cell division.

Portal vein embolisation (PVE) is an established technique to achieve curative resection in patients who would otherwise be advised against surgery due to an anticipated inadequate residual liver volume. The resection rate after PVE is 60–70%, mainly limited by disease progression (Mueller *et al*, 2008; Pamecha *et al*, 2009). This may reflect either a rapid disease progression in patients who are selected for PVE or that PVE stimulates tumour growth. Tumour growth exceeding that of the normal liver parenchyma has been shown on imaging following PVE in patients with contra lateral lobe metastases (Elias *et al*, 1999), and the tumour-doubling time following PVE has been shown to be reduced from 92 to 76 days (Kokudo *et al*, 2001). There have been concerns that PVE may increase the risk of disease recurrence after curative resection (Kokudo *et al*, 2001; Pamecha *et al*, 2009). The influence of PVE on tumour growth characteristics on resected cancers has not been investigated. This study was designed to evaluate the effect of PVE on tumour growth and cancer-cell proliferation in patients with resected colorectal liver metastases.

## Materials and methods

The study involved 22 patients who had undergone liver resection and had required PVE due to an inadequate anticipated residual liver volume. Over the study period (September 1999 to September 2005), 109 patients underwent major liver resection (⩾3 liver segments) for colorectal cancer (CRC) metastases at the centre. Eighty-seven of these patients underwent liver resection without PVE. Twenty of these 87 patients had similar tumour volumes at presentation compared with the patients undergoing PVE and were therefore selected as a non PVE control group. Clinical details of the patients were collected prospectively, including demographics, stage of the primary disease, details of the liver metastases and chemotherapy received.

### Volumetric measurements and tumour growth rate analysis

Tumour volumes were measured by CT (GE Medical Systems High Speed System, Milwaukee, WI, USA) or MRI (Philips Intera 1 T System Philips, Netherlands) at the time of diagnosis. The technique for measuring volumes has been described earlier (Beal *et al*, 2006). Briefly, axial 10-mm sections through the liver were obtained in a single breath-hold. Using a workstation, the tumour volume was calculated by multiplying the area of each liver image by the slice thickness. An experienced hepato-biliary radiologist performed all the measurements. The tumour volume was also calculated from the resected specimen by measuring the three dimensions of the tumour and calculating the volume. Tumour growth was calculated (ml day^–1^) by subtracting the tumour volume at diagnosis from the volume at resection and dividing by time in days. The growth rate between the two groups was compared.

### Histological analysis

Resected specimens were sliced and fixed in 10% formalin for 24 h. Protocol samples were paraffin processed and tissue sections were prepared using routine laboratory standard procedures and stained with haematoxylin and eosin. The predominant cellular differentiation and the percentage of necrosis were identified. The mitotic index and apoptotic counts were calculated in 10 high-power fields of viable tumour.

### Immunohistochemistry

Proliferative activity of the tumours was assessed by using the mouse antihuman monoclonal *Ki67* antibody (clone MIB-1) obtained by DAKO (Glostrup, Denmark). The antibody reacts with human *Ki67* nuclear antigen which is only expressed during cell division. Sections of formalin-fixed, paraffin-embedded tissues (3 mm) were cut into charged slides and sections were immunostained as per protocol (microwave antigen retrieval in ethylene diamine tetraacetic acid (EDTA) buffer for 20 min at 850 W, antibody concentration 1 out of 300, Novolink Polymer Detection System, Novocastra Vision Byosystems, UK). The labelling index was determined by using a Glasgow cell-counting graticule (Going, 1994). Areas where labelling was the highest were chosen and necrotic areas were avoided. The number of *Ki67*-positive cells was counted in 10 high-power fields and expressed as a percentage of the total number of cancer cells. Histological assessment was carried out by two experienced pathologists (A Levene and F Grillo) independently, who were blinded to the treatment group and related clinical data.

### Chemotherapy

Of the 22 patients undergoing PVE, all had chemotherapy before and after the procedure. Chemotherapy post-PVE was started 2 weeks after embolisation and continued for 6 weeks or until a decision was made regarding liver resection surgery. All 20 patients in the control group received chemotherapy before the liver resection. Three patients with PVE received chemotherapy after liver resection because of involved (*n*=1) or close (*n*=2) resection margin (13.6%). Two patients without PVE received chemotherapy after liver resection because of involved (*n*=1) or close (*n*=1) resection margin (10%). In all cases, this comprised a standard 5FU and folinic-acid-based protocol combined with oxaliplatin or irinotican. The average number of cycles was six.

### Statistics

Student *t*-tests were used to compare the data. Data are expressed as means with 95% confidence intervals and median with ranges. *P*<0.05 was considered significant. Survival rates were calculated using the Kaplan–Meier methods.

## Results

The patient, primary tumour and liver metastases characteristics are shown in Table 1. The groups were well matched by age, sex, primary tumour site, Duke's stage and the temporal relationship of the liver metastases (synchronous/metachronous) to the presentation of the primary.

**Figure 1:**
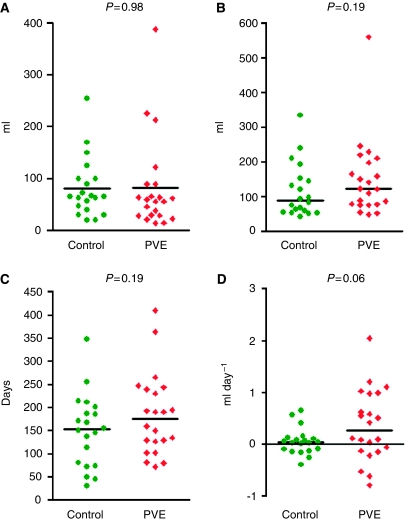
(**A**) Tumour volume at diagnosis. (**B**) Tumour volume at resection. (**C**) Time difference from diagnosis to resection. (**D**) Tumour growth rate.

As would be expected the volumes of liver metastases at presentation in the groups were the same (control: 81.41±57, PVE: 81.3±88, *P*=0.98 (Figure 1A)). The tumour volume at resection was higher in the PVE group, but this was not significant (control: 116±77, PVE: 149±110, *P*=0.19) (Figure 1B). The time from presentation to resection was longer in the PVE group by an average of 33 days (183±88 *vs* 150±78, *P*=0.19) (Figure 1C). The tumour growth rate (ml day^–1^) was more rapid in the PVE group compared with controls (control: 0.05±0.25, PVE: 0.36±0.68, *P*=0.06) (Figure 1D).

**Figure 2:**
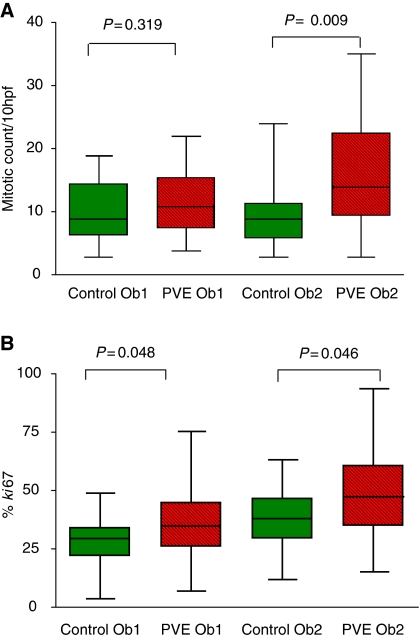
Mitotic count and *Ki*67 by two independent pathologists. (**A**) Mitotic count. (**B**) *Ki*67 count.

**Figure 3:**
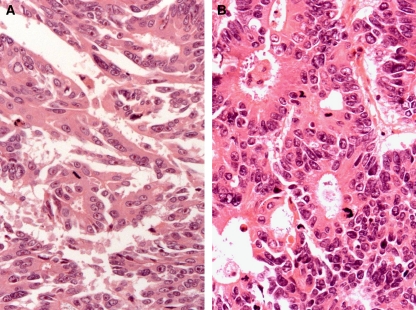
Typical example of mitosis expression (dark brown stain) in CRC metastases for a patient who had not undergone PVE (**A**) compared with that post PVE (**B**), suggesting high cancer-cell proliferation post PVE.

**Figure 4:**
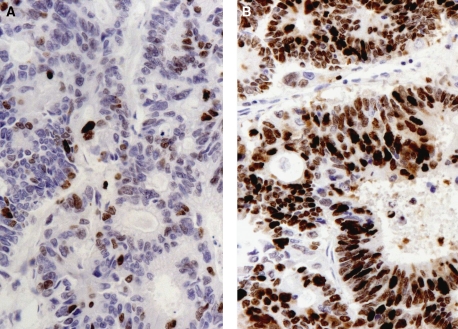
Typical example of *Ki*67 expression (dark brown stain) in CRC metastases for a patient who had not undergone PVE (**A**) compared with that post PVE (**B**), suggesting high cancer-cell proliferation post PVE.

The morphology of the resected tumours in both groups was evaluated by assessing tumour differentiation, necrosis and apoptosis. These variables were not different between the groups (Table 2). The mitotic rate (Figure 2A) was increased following PVE as measured by both histopathologists, although statistically significant for only one (Table 2). The *Ki67* proliferation index increased significantly following PVE (Table 2) (Figures 2B, 3 and 4).

A subgroup analysis was carried out comparing the tumour growth and *Ki67* in patients with synchronous and metachronous metastases (Table 3). Growth rate and proliferation index were increased by PVE in both groups, but was statistically significant only in the metachronous group.

**Figure 5:**
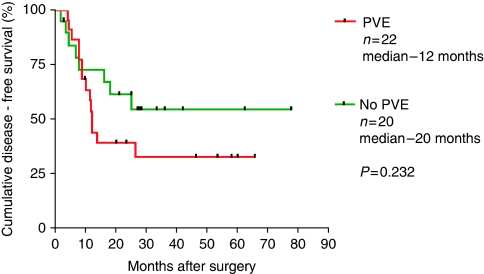
Disease-free survival after liver resection for colorectal metastases with and without prior PVE.

**Figure 6:**
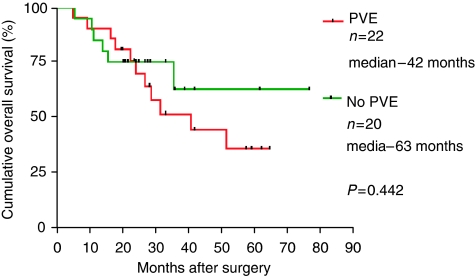
Overall survival after liver resection for colorectal metastases with and without prior PVE.

The 5 year survival of the patient group who had undergone PVE was 25% compared with 55% for the control group (*P*=ns) (Figure 5).The median disease-free survival of patients undergoing liver resection following PVE was 12 months, and 24 months in those resected without undergoing PVE (Figure 6).

## Discussion

Liver resection provides the main possibility of cure in patients with colorectal liver metastases and 5-year survival ranges from 25 to 58% (Simmonds *et al*, 2006). One of the contraindications to hepatic resection is a small future liver remnant. The small residual liver volume can lead to cholestasis, fluid retention, impaired liver synthetic function and liver failure (Melendez *et al*, 2001; Schindl *et al*, 2005). PVE increases the anticipated future liver volume and allows surgery in patients who would otherwise be contra-indicated for resection. The potential disadvantage of PVE is that earlier studies have suggested that it may stimulate tumour growth and lead to reduced long-term survival (Elias *et al*, 1999; Kokudo *et al*, 2001).

This study has examined the effect of PVE on tumour growth and morphology. The tumours grew significantly during the period between diagnosis and resection. The growth rate was significantly higher following PVE. The control group was well matched by tumour stage and burden;although the period between initial diagnosis and resection was longer for the PVE group (by 33 days), this was not statistically significant. This would suggest that PVE stimulates tumour growth. Any difference in the growth rates of the two groups could have been more effectively addressed by including a comparison of growth rates before PVE. However, interim CT scans were not available in the control group . Another possible explanation for the tumour volume changes following PVE would be ischemia and inflammation rather than cancer growth. However, the resected cancers showed no evidence of haemorrhage and the degree of cell necrosis and apoptosis was similar with and without PVE.

This is the first study to compare tumour growth from imaging with increased cancer-cell proliferation at the molecular level following PVE. The mitotic index is a sensitive but non specific marker of cell proliferation (Peeters *et al*, 2006). The mitotic index was increased in blinded assessment by both histopathologists but was statistically significant for one observer only. This inter-observer variation in the mitotic index can be explained by difference in appreciation of dividing cells by the two histopathologists. The *Ki67* has been shown to be more specific in assessing the cancer-cell proliferation rate (Peeters *et al*, 2006). The *Ki67* labelling index was significantly higher in the PVE group than controls. A high *Ki67* labelling index is an adverse prognostic factor in patients undergoing hepatectomy for colorectal liver metastases (Weber *et al*, 2001). Identification of the *Ki67* labelling index before and after PVE in the same patient would have been of interest, but pre-PVE biopsy was not carried out for ethical reasons as it carries a risk of tumour seeding.

A subgroup analysis of patients with synchronous and metachronous metastases showed tumour growth rate and *Ki*67 to be greater post PVE for the latter. The reason for this has not been established and should be the subject of further study. It is possible that there is a biological difference between these tumours which allows PVE to have a more marked effect on growth with metachronous disease.

The increase in tumour growth following PVE could be secondary to haemodynamic changes. Portal vein embolisation increases hepatic arterial blood flow (Nagino *et al*, 1998; Denys *et al*, 2000; Yokoyama *et al*, 2007). As intrahepatic metastases depend solely on arterial blood supply (Archer and Gray, 1989), increased hepatic arterial flow may provide nutritional advantages for tumour growth. A correlation between tumour growth rate and arterial flow to the liver could clarify these hypotheses.

Tumour growth could be initiated by a reduction in cell apoptosis or increase in cell division (Peeters *et al*, 2006). Several cytokines and growth factors are known to play important roles in liver regeneration (Kusaka *et al*, 2006; Yokoyama *et al*, 2007), and could increase tumour growth after PVE. Mueller *et al*, reported, in a rat model of portal branch ligation, an association between hepatic atrophy and increased expression of genes known to promote tumour growth and angiogenesis (Mueller *et al*, 2003). Expression of hepatocyte growth factor (HGF)-mRNA is markedly increased after portal vein ligation (Uemura *et al*, 2000), which is known to stimulate growth of colorectal carcinoma cells *in vitro* (Ueno *et al*, 1996; Nabeshima *et al*, 1998). Negative regulators of hepatocyte proliferation, such as transforming growth factor (TGF)-*β*1 (Braun *et al*, 1988; Kusaka *et al*, 2006), are strongly expressed in the ligated lobe (Uemura *et al*, 2000; Kusaka *et al*, 2006) and these may contribute to increased cancer-cell proliferation.

The survival analysis showed excellent long-term survival in patients undergoing major liver resection for CRC metastases with a 55% 5-year survival for the control (no PVE) group. This figure compares favourably with reports from other centres suggesting a high-quality oncological surgery (Simmonds *et al*, 2006). The PVE group, despite having resection with clear margins, had a lower disease-free and long term survival. As the patient groups were well matched for cancer stage, this would strongly support the molecular evidence that PVE stimulates cancer cell division and, as a result, is associated with a reduced long term outcome.

In the present study, tumour growth was observed in patients following PVE despite pre- and post-PVE chemotherapy. Although pre- and-post operative chemotherapy may provide a small survival advantage, this should be short course and should avoid delaying surgical intervention (Nordlinger *et al*, 2007). In the light of increased hepatic arterial flow following PVE, which may be contributing to tumour growth, there is a logical reason for anti-angiogenic agents, such as Bevacizumab, along with routine chemotherapy (Hurwitz *et al*, 2005; Saltz *et al*, 2007) to cover the peri PVE period. This possibility requires evaluation in a prospective study.

In conclusion, although PVE appears to benefit patients by facilitating liver resection in those who would be considered inoperable because of insufficient future liver remnant volume, there are concerns of stimulated tumour growth and inferior long term survival. Patients for PVE should be selected carefully and PVE should be avoided in patients with an adequate future liver remnant. At present, selection of patients for PVE is based on CT/MRI volumetry, and 30% of future liver remnant volume is considered adequate in patients with normal liver and 40% in patients with abnormal liver function (Abdalla *et al*, 2001). Incorporating hepatic functional studies, such as hepatic scintigraphy (Dinant *et al*, 2007) and biopsy of normal liver, to evaluate histological abnormality (steatosis, steatohepatitis and cholestasis) in patients with borderline future liver remnant will provide further information on the quality of the residual liver and may avoid unnecessary PVE.

## Figures and Tables

**Table 1:** Demographics, primary tumour and liver metastases characteristics

	**Control (*n*=20)**	**PVE (*n*=22)**	***P*-value**
Age (range)	61.5 (39–78)	62.5 (46–78)	0.876
Female	9	10	0.724
Dukes (B/C)	4/16	5/17	0.754
Rectal/colon	6/14	9/13	0.213
Syn/Meta	10/10	13/9	0.537
Ext Rt / Rt Hep	8/12	14/8	0.548
Number of tumours	2 (1–5)	3 (1–9)	0.153
Resection margin (mm)	20.2±3.7	14.7±4.2	0.338
Number of chemotherapy cycles	6 (3–10)	6 (3–10)	0.141

Syn=synchronous; Meta=metachronous; Ext Rt=extended right hepatectomy; Rt Hep=right hepatectomy; PVE=portal vein embolisation.

**Table 2:** Tumour morphology with and without PVE

	**Control (*n*=20)**	**PVE (*n*=22)**	***P*-value**
*Necrosis (%)*			
Median	35 (15–95)	45 (10–95)	
Mean±s.d.	39.5±4.9	37.7±4.8	0.801
			
*Apoptosis/10 hpf*			
Median	188 (54–327)	132 (68–350)	
Mean±s.d.	178±87	147±69	0.212
			
*Mitotic count/10 hpf*			
Observer 1			
Median	9 (3–19)	11 (4–22)	0.404
Mean±s.d.	10.5±1.0	11.8±1.1	
Observer 2			
Median	9 (3–24)	14 (3–35)	
Mean±s.d.	9.7±5.0	16.1±8.8	0.009
			
*Ki67 (%)*			
Observer 1			
Median	29 (3–48)	36 (6–75)	
Mean±s.d.	22.6±2.2	36.7±3.8	0.048
Observer 2			
Median	38(11–62)	47(15–93)	
Mean±s.d.	37.8±13.8	48.9±18.9	0.046

PVE=portal vein embolisation.

**Table 3:** Subgroup analysis of tumour growth rate and *Ki*67 for synchronous and metachronous colorectal liver metastases

	**Control**	**PVE**	***P*-value**
*Tumour growth rate (ml day* ^–1^ *)*			
Metachronous	*n*=10	*n*=9	
Median	−0.08 (−0.57–0.07)	0.42 (−1.2–2.0)	
Mean±s.d.	−0.15±0.22	0.41±0.88	0.06
Synchronous	*n*=10	*n*=13	
Median	0.09 (−0.26–0.66)	0.51 (−1.1–1.0)	
Mean±s.d.	0.01±0.26	0.28±0.59	0.15
			
*Ki 67(%)*			
Metachronous	*n*=10	*n*=9	
Median	21.85 (3.8–35.8)	37.40 (6.9–75)	
Mean±s.d.	21.8±10.5	41.5±20.4	0.01
Synchronous	*n*=10	*n*=13	
Median	31.9 (28.1–48.5)	34 (11.9–61)	
Mean±s.d.	33.3±6.1	34±13.4	0.89

PVE=portal vein embolisation.
